# The roles of the human ATP-binding cassette transporters P-glycoprotein and ABCG2 in multidrug resistance in cancer and at endogenous sites: future opportunities for structure-based drug design of inhibitors

**DOI:** 10.20517/cdr.2021.19

**Published:** 2021-08-04

**Authors:** Jason Goebel, Jean Chmielewski, Christine A. Hrycyna

**Affiliations:** Department of Chemistry, Purdue University West Lafayette, IN 47907, USA.

**Keywords:** P-glycoprotein, ABCG2, ABC transporters, multidrug resistance, inhibitor

## Abstract

The ATP-binding cassette (ABC) transporters P-glycoprotein (P-gp) and ABCG2 are multidrug transporters that confer drug resistance to numerous anti-cancer therapeutics in cell culture. These findings initially created great excitement in the medical oncology community, as inhibitors of these transporters held the promise of overcoming clinical multidrug resistance in cancer patients. However, clinical trials of P-gp and ABCG2 inhibitors in combination with cancer chemotherapeutics have not been successful due, in part, to flawed clinical trial designs resulting from an incomplete molecular understanding of the multifactorial basis of multidrug resistance (MDR) in the cancers examined. The field was also stymied by the lack of high-resolution structural information for P-gp and ABCG2 for use in the rational structure-based drug design of inhibitors. Recent advances in structural biology have led to numerous structures of both ABCG2 and P-gp that elucidated more clearly the mechanism of transport and the polyspecific nature of their substrate and inhibitor binding sites. These data should prove useful helpful for developing even more potent and specific inhibitors of both transporters. As such, although possible pharmacokinetic interactions would need to be evaluated, these inhibitors may show greater effectiveness in overcoming ABC-dependent multidrug resistance in combination with chemotherapeutics in carefully selected subsets of cancers. Another perhaps even more compelling use of these inhibitors may be in reversibly inhibiting endogenously expressed P-gp and ABCG2, which serve a protective role at various blood-tissue barriers. Inhibition of these transporters at sanctuary sites such as the brain and gut could lead to increased penetration by chemotherapeutics used to treat brain cancers or other brain disorders and increased oral bioavailability of these agents, respectively.

## INTRODUCTION

Multidrug resistance (MDR) in cancer is a complex, multifactorial problem in which tumors are either intrinsically resistant to chemotherapeutic treatment or acquire resistance to drugs over the course of treatment. Cancer cells can develop MDR through various mechanisms, including drug sequestration in intracellular compartments, downregulation of cell death mechanisms, microenvironmental changes, and expression of ATP-binding cassette (ABC) transporters^[1-9]^. Unlike other types of resistance that alter the effects of only one drug, ABC transporter expression confers cellular resistance to a wide variety of chemically and structurally unrelated anti-cancer agents, a phenomenon known as MDR^[3,6,10-13]^. Most notably, the expression of ABCB1, commonly known as P-glycoprotein (P-gp), and ABCG2, also known as BCRP or the breast cancer resistance protein, in tumor cells has been correlated to poor patients prognosis in numerous studies. However, it was unclear whether these correlations were causal since expression levels in patient samples did not reach those observed in cultured cells^[14-17]^. Although overexpression of other ABC transporters, including MRP1/ABCC1, has also been implicated in cancer MDR^[18]^, this review focuses on P-gp and ABCG2. Though found in different subfamilies of the ABC transporter superfamily, these two transporters have similar overlapping substrate and inhibitor specificities. This knowledge, coupled with recent structures of both ABCG2 and P-gp with inhibitors bound, offer the opportunity to explore the future of rational structure-based drug design of either specific or dual inhibitors for the reversal of MDR clinically or for use in delivering therapeutic agents to sanctuary sites in the body protected by the endogenous expression of these transporters.

Given this clinical correlation, it was hypothesized, in retrospect naïvely so, that inhibition of these transporters would result in the reversal of the MDR phenotype clinically in patients. As such, over the past three decades, three generations of agents have been developed that showed great promise as potent inhibitors in *in vitro *and some *in vivo *model systems^[19-24]^. However, no inhibitor to date has been shown to significantly reverse MDR in human clinical trials^[17,24-29]^. For a comprehensive list and discussion of these human clinical trials, please see references^[17,23,26,29-32]^. This lack of success and a greater understanding of the molecular complexity of MDR in tumors has led to substantial and, to a great extent, warranted skepticism in the field regarding whether this approach will ever be successful in reversing MDR in patients^[17,24,28,29,33]^.

However, as is discussed more in-depth herein, these trials were flawed in many ways, including poor patient selection, a lack of understanding of the different subtypes of cancers within a patient population, the presence of other mechanisms of MDR, the effects of expression of other transporters in the cells and P-gp transporter polymorphisms^[17,24,29]^. Coupled with the lack of high-resolution structures of the transporter with substrates or inhibitors, which severely limited rational drug design efforts for P-gp and ABCG2, enthusiasm for this approach waned considerably. However, recent advances in structural biology, especially in cryo-EM, have resulted in high-resolution three-dimensional structures of P-gp and ABCG2^[34]^, allowing for a complete dissection of their molecular mechanisms. As discussed in detail herein, the emergence of the high-resolution structures of these transporters in complex with inhibitors should finally allow for true rational structure-based drug design of more potent inhibitors with enhanced specificity. In turn, better inhibitors and future rigorously designed clinical trials specifically targeting patient populations with tumors expressing P-gp, may offer more promising results in some subsets of cancer patients^[17,29]^. Other methods such as controlling ABC transporter expression through microRNAs and small interfering RNAs (siRNAs) and monoclonal antibodies are also currently being researched as possible ways to inhibit P-gp and ABCG2^[35]^.

P-gp and ABCG2 are endogenously expressed at the blood-brain barrier (BBB) and the intestinal epithelium, where they serve a protective role in limiting the entry of xenobiotics into the brain and bloodstream respectively^[36-38]^. However, during chemotherapeutic treatment, this protective role can be thought of as a form of drug resistance since the transporters limit the number of drugs that can reach target cancer cells. Therefore, even if inhibitors of P-gp and ABCG2 do not ultimately prove useful for MDR cancers directly, they may prove to be efficacious as tools to increase the bioavailability of chemotherapeutic agents to sites protected by endogenously expressed ABC transporters, such as the central nervous system and the gut.

## ABC TRANSPORTERS AND CULTURED CELL MULTIDRUG RESISTANCE

The failure of chemotherapy in cancer is often due to either intrinsic or acquired resistance to many structurally and chemically unrelated compounds over the course of the treatment, a phenomenon called MDR^[1-3]^. One mechanism studied extensively is ABC transporters’ expression limiting the number of drugs that can enter cells by effluxing the agent from the cells in an ATP-dependent manner^[3,6-9,25]^. This phenotype was initially described in a subculture of HeLa cells grown in selective media containing actinomycin D^[39]^. Several Chinese hamster ovary (CHO) cell lines cultured with increasing concentrations of actinomycin D were also shown to be resistant to some other drugs, including daunomycin, vincristine, and vinblastine^[40]^. In further studies of the nature of this acquired resistance, Danø demonstrated active transport of actinomycin D in drug-resistant Erlich ascites tumor cells, leading to the hypothesis that the protein responsible for the MDR phenotype was a transporter^[41]^. This transporter was subsequently identified in CHO cells that were shown to be resistant to some unrelated drugs^[42]^. Due to observed glycosylation and its role in reducing the permeability of many unrelated drugs in resistant cell lines, the 170 kDa transporter was given the name permeability glycoprotein or, as it is more commonly referred, P-glycoprotein or P-gp^[42]^. P-gp was subsequently purified from plasma membrane-derived vesicles of colchicine-resistant CHO cells^[43]^. The *mdr* gene encoding P-gp was first cloned from colchicine-resistant CHO cells and soon after in human KB carcinoma cells^[44-46]^. Since the discovery of P-gp, an entire superfamily of transporters has been discovered and continues to be studied^[47-52]^. P-gp is a member of this family of ABC transporters and has been given the gene classification of ABC subfamily B member 1, ABCB1^[47]^. More recently, another member of the ABC superfamily that has been shown to confer multidrug resistance in cancer cells in culture is the BCRP or ABCG2. ABCG2 was discovered in adriamycin-resistant MCF-7 breast cancer cells and mitoxantrone-resistant S1-M1-80 colon carcinoma cells as well as in the placenta, where it is endogenously expressed^[53-55]^. Further study of the ABC transporter superfamily revealed 48 human membrane transporters with diverse functions^[47,56]^.

## CLINICAL TRIALS

The results of the cell culture model studies and studies correlating P-gp expression to poor clinical outcome fueled great excitement for the potential therapeutic use of P-gp inhibitors to reverse MDR clinically^[17,29]^. As a result, numerous clinical trials were performed to determine if adding a P-gp inhibitor to drug regimens would improve therapeutic efficacy. Unfortunately, many inhibitors of P-gp and ABCG2 that were successful *in vitro* did not prove to be efficacious in these human trials^[3,17,19,27,29,57,58]^. However, many of these clinical studies were flawed from the outset in significant ways^[3,17,27,29,58]^. Perhaps most importantly, in most trials, patients were not selected for inclusion in the study based on tumor expression of P-gp^[17,58]^.

More recently, robust analyses of drug-resistant tumors using new genomic tools and diagnostic methods suggest that resistance caused by P-gp, ABCG2, and perhaps other ABC transporters may be limited to subsets of tumors and/or subsets of patient populations. Further developments in flow cytometry, positron emission tomography (PET), FISH analysis, RNA-seq, and next generation sequencing, will more readily allow for further in-depth evaluation of samples that may aid in selecting the subset of patients who would most likely respond to the use of a P-gp or ABCG2 inhibitor. In a genome-wide characterization of chemoresistant ovarian carcinoma patients, it was shown that only a small subset demonstrated the recurrent promoter fusion event associated with P-gp overexpression^[59]^, suggesting, perhaps, that this subset of patients might benefit from this clinical approach rather than of the entire cohort. In a recent study where flow cytometry was used to determine P-gp expression in the bone marrow and peripheral blood samples from 346 patients diagnosed with AML, the patients were further stratified based on age, gender, and disease status^[60]^. Although overall, P-gp was expressed in only 32.1% of patients, a greater prevalence of P-gp expression was found in older patients and those with the refractory, recurrent and secondary disease as compared to *de novo* AML. While P-gp expression was only found in 60/261 (22.9%) of *de novo* AML patients, it was found in 23/24 (95.8%) of patients with the drug-refractory disease and 24/30 (80.0%) of those with relapsing AML^[60]^. Positron emission tomography (TEM) is another powerful tool used to detect and/or measure the expression of P-gp using radio-labeled substrates and inhibitors. PET was first used to measure P-gp function at the BBB non-invasively in 1998 in *mdr1a(-/-)* knockout mice with [^11^C]verapamil and cyclosporin A^[61,62]^. Since this initial work, PET has also been used to evaluate whether alterations of P-gp function at the BBB are implicated in the pharmacoresistance of some HIV and epilepsy patients to therapeutics or if P-gp function is either compromised or enhanced in several other central nervous system disorders^[62]^. In total, although the results of many clinical trials generally dampened enthusiasm tremendously for a P-gp inhibitor approach to treating MDR cancers, in the new era of personalized medicine and advanced diagnostic techniques as described above, it is worthwhile to repeat these trials with more stringent requirements for inclusion and the use of more specific and more potent inhibitors.

## ENDOGENOUS EXPRESSION OF P-gp AND ABCG2: LIMITING ORAL BIOAVAILABILITY AND DRUG PENETRATION OF THE CNS

As described above, ABC transporters present in the plasma membrane of multidrug-resistant cancer cells may be responsible for significant alterations in the pharmacokinetics and efficacy of many chemotherapeutics^[3,6,10-13]^. Additionally, endogenous expression of P-gp and ABCG2 transporters at blood-tissue barriers is known to lead to reduced uptake of therapeutics across these barriers^[11,36-38,63-66]^. In our view, the limited bioavailability of therapeutics to these sanctuary sites constitutes an alternate form of MDR that, if overcome, could result in more efficacious treatments of not only cancer but other diseases.

At the BBB, the ABC transporters P-gp and, to a somewhat lesser extent, ABCG2 are thought to serve a protective function by limiting the accumulation of lipophilic agents in the brain by effluxing the compounds back into the bloodstream^[36,67-69]^. To probe if P-gp and ABCG2 limit the accumulation of chemotherapeutic agents in the brain, P-gp-null, and ABCG2-null mice have been studied^[11,37,69-76]^. Using a mouse knockout model, P-gp was found to be an important determinant in the brain penetration and pharmacological activity of many drugs^[11,69]^. For example, many of the chemotherapeutic agents used to treat glioblastomas are, in fact, P-gp or ABCG2 substrates, including Gleevec, topotecan, and paclitaxel^[74,77,78]^. Another study demonstrated *in vivo* and *in vitro* that co-administration of paclitaxel with the potent P-gp inhibitor valspodar (PSC833) increased the levels of fluorescently labeled paclitaxel in normal mouse brains^[79]^. Furthermore, it was demonstrated that co-administration of these two drugs reduced the growth of implanted human glioblastomas by 90% in nude mice, whereas treatment with paclitaxel or valspodar alone did not affect tumor size^[79]^.

In ABCG2 knockout mice, increased brain accumulation of drugs has been observed when only ABCG2 was eliminated, especially for compounds that are not substrates for both transporters^[69,78,80]^. Interestingly, however, synergistic effects on drug brain accumulation are observed in double ABCG2/P-gp knockout mice for drugs that are substrates of both transporters, further enforcing their functional redundancy *in vivo*^[78,81-83]^. For example, whereas the brain concentration of the kinase inhibitor ceritinib was approximately 38-fold higher in P-gp-only knockout mice compared to wild-type, the concentration increased to 90-fold in brains of mice lacking both P-gp and ABCG2^[82]^. Another study demonstrated an approximately 20-fold increase of vemurafenib in the brains of P-gp/ABCG2 knockout mice compared to no increase in an ABCG2 knockout and a 1.7-fold increase in a P-gp knockout^[81]^. Thus, although P-gp appears to often be the dominant ABC transporter at the blood-brain barrier, these data suggest that dual P-gp/ABCG2 inhibitors may have even greater therapeutic promise for brain diseases^[23,84-86]^.

P-gp and ABCG2 are also expressed endogenously on the apical side of the intestine and colon epithelial cells, where they are thought to protect from ingested xenobiotics^[87-89]^. Given this localization, these transporters have also been implicated in limiting the bioavailability of many oral therapeutics^[38,90,91]^. For example, studies in P-gp *mdr1a(-/-)* knockout mice showed an approximate 25% increase in the oral bioavailability of paclitaxel as compared to wild-type mice^[92]^. Furthermore, in wild-type mice, co-administration of elacridar (GF120918) with paclitaxel increased the plasma values for orally dosed paclitaxel in wild-type mice by 6.6-fold. Importantly, these results were also observed in the plasma of patients treated with paclitaxel combined with elacridar or cyclosporin A^[93]^.

Similarly, the uptake of therapeutics, such as topotecan and quercetin, has been shown to be limited in ABCG2 homozygous knockout mice and rats^[94,95]^. In addition, the co-administration of gefitinib, an ABCG2 inhibitor, has also been shown to increase the oral uptake of sulfasalazine by 13-fold compared to sulfasalazine alone in a mouse model^[96]^. These data together suggest that inhibition of P-gp and ABCG2 in the gastrointestinal tract is a viable method for increasing the bioavailability of oral chemotherapeutic agents. However, as the pharmacokinetic and pharmacodynamic properties of the agents will be altered upon systemic inhibition of P-gp, whole-body toxicity and especially toxicity at sanctuary sites protected by endogenous P-gp, such as the brain, must be considered. Thus, the dose of the therapeutic agent would necessarily have to be optimized, and likely lowered to maximize drug exposure but minimize whole-body toxicity.

## P-gp AND ABCG2 STRUCTURE AND MECHANISM

P-gp and ABCG2, members of the ABC transporter superfamily, are transmembrane proteins that use the energy of ATP to efflux substrates across a membrane^[13,34,48,97,98]^. Structurally, P-gp and ABCG2 consist of four common domains to the ABC transporter superfamily, although many other ABC transporters also contain additional auxiliary domains or only nucleotide-binding domains^[47]^. Both P-gp and ABCG2 transporter have two cytoplasmic nucleotide-binding domains (NBDs) that hydrolyze ATP and two transmembrane domains (TMDs) that recognize and bind the substrate molecules and form a pathway for their translocation across the membrane^[34,97,99]^. These domains can be encoded by a single gene that produces a full-length transporter, like P-gp, or by two genes that encode a half-transporter with one NBD and one TMD that homodimerize to produce a full-length transporter, like ABCG2^[34,47,48]^. In addition, some full-length ABC transporters are heterodimers of two distinct half-transporters (e.g., ABCG5/ABCG8). The NBDs are highly conserved across the superfamily and fall into the P-loop NTPase superfamily with a RecA-type binding core along with a unique ABC signature motif, LSGGQ^[34]^. The TMDs are far less conserved, allowing for a range of substrates to be recognized^[47]^.

**Figure 1:**
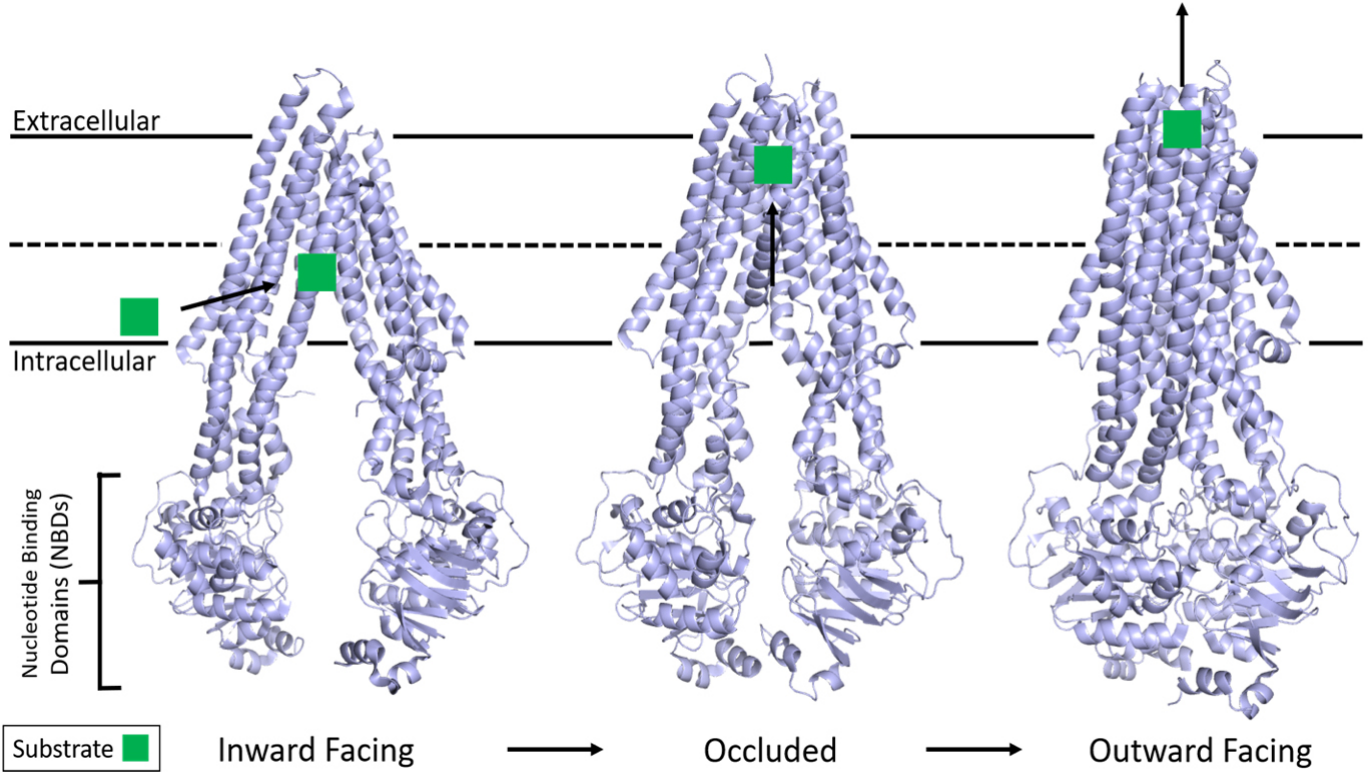
Structure of P-gp and the basic mechanism of ATP hydrolysis and transport. P-gp undergoes large conformational changes in its transport mechanism. The membrane is delineated by solid black lines. Substrate from the inner leaflet first interacts with an inward-facing conformation of P-gp while two ATP molecules bind the free NBDs. The structure then adopts an occluded state with substrate bound at the apex of the TMDs while TM helices 4 and 10 significantly kink inwards, occluding the binding pocket. The outward-facing conformation is correlated with asymmetric ATP hydrolysis and solvent-exposed substrate diffusion into extracellular space before ADP release and the mechanism resetting. Adapted from PDB: 5KPI (inward)^[94]^, 7A6C (occluded)^[96]^, and 6C0V (outward)^[95]^. NBDs: Nucleotide-binding domains; TMDs: transmembrane domains.

As informed first by a plethora of past biochemical and pharmacological experiments^[13,100-109]^, and currently by the three-dimensional structures^[110-121]^, the basic scheme of ATP-dependent substrate transport in P-gp involves large conformational changes during the efflux cycle [Figure 1]. The hydrophobic P-gp substrates diffuse into the membrane and interact with the transporter via the inner leaflet. Substrate binding signals P-gp to adopt a state in which substrate is bound at the apex of the TMDs, creating an occluded binding pocket^[112]^. Interestingly, this study also showed that while only one molecule of the substrate vincristine was able to bind, inhibitors such as elacridar, tariquidar, or zosuquidar were bound in pairs.

It is clear that the energy from ATP hydrolysis is required to complete a substrate transport cycle and that, although both NBDs are catalytically active, they are functionally asymmetric, with only one hydrolysis event occurring at a time^[107-109,122]^. For human P-gp, the three-dimensional structure demonstrated that ATP binding drives dimerization of the NBDs, causes the transition to the outward-facing conformation, and promotes subsequent substrate release^[111]^. However, recent double electron-electron resonance electron paramagnetic resonance (DEER EPR) experiments with P-gp demonstrated that substrate transport could not proceed until at least one ATP is hydrolyzed^[123]^. It has been further postulated that ATP hydrolysis and/or subsequent Pi or ADP release is required for resetting the transporter back to the inward-facing conformation to accept another substrate molecule^[34,111]^. Interestingly, the basal ATP hydrolysis observed in P-gp is hypothesized to be an intrinsic property that allows it to sample a multitude of conformations as a mechanism to recognize a diverse set of substrates^[110]^.

Concurrently, the structures of human ABCG2 in various states have also been solved^[124-127]^. Unlike P-gp, ABCG2 does not show large conformational changes during the transport cycle, as the NBDs are in close proximity in the nucleotide-free state. In ABCG2 structures, ATP binding has been shown to cause the NBDs to dimerize more closely, which, in turn, collapses the inward-facing drug-binding cavity and opens the outward-facing cavity to the extracellular space^[125]^. Further, no occluded intermediate conformation in the presence of substrate was observed for ABCG2. As such, it is proposed that ATP binding alone might be sufficient to move the substrate to the external cavity and subsequent release. Further, it is proposed that ATP hydrolysis and/or subsequent Pi or ADP release would be required for resetting the transporter. At present, neither the structural nor functional reason for the proximity of the NBDs in ABCG2 compared to P-gp has been elucidated.

## P-gp AND ABCG2 SUBSTRATE AND INHIBITOR BINDING SITES

Due to the promiscuous and adaptive nature of the drug binding sites of both P-gp and ABCG2, these transporters are able to recognize, bind, and transport a wide variety of substrates. For example, P-gp recognizes many unrelated neutral and cationic hydrophobic anti-cancer agents, including anthracyclines, camptothecins, epipodophyllotoxins, taxanes, tyrosine kinase inhibitors, and vinca alkaloids in cell culture models^[13]^. Substrate recognition by ABCG2 has overlap with P-gp, but with some notable differences, including transport of the anti-cancer drugs flavopiridol, irinotecan, methotrexate, and mitoxantrone^[128,129]^. In addition, numerous biochemical studies have demonstrated the existence of at least three spatially distinct substrate binding sites within the transmembrane regions of P-gp that function in substrate transport or regulation of substrate transport^[130-141]^. In total, these studies revealed a mostly hydrophobic region of residues from multiple transmembrane domains that are capable of binding not only a large variety of substrates but also are able to bind multiple substrates simultaneously.

**Figure 2:**
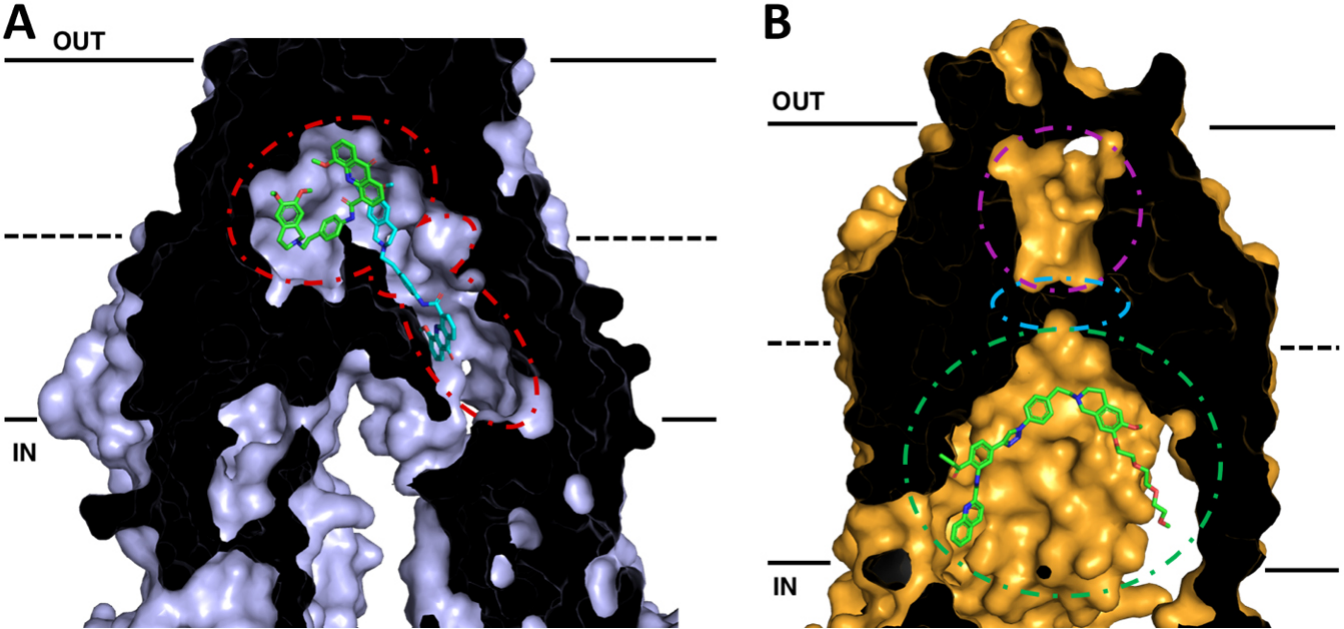
Cross-sections of cryo-EM structures of P-gp and ABCG2 with bound inhibitors in inward-facing conformations. The membrane is delineated by solid black lines. The extracellular space (OUT) is at the top of the image, and the intracellular space (IN) is at the bottom. (A) Cryo-EM structure of P-gp bound with two molecules of the inhibitor elacridar. One molecule (green) is bound to the drug-binding pocket while the other (cyan) passes through a smaller binding region, the vestibule, before extending into an access tunnel. (B) Cryo-EM structure of ABCG2 with bound the tariquidar analog MB136. MB136 is bound to the top of cavity 1 (green) under the leucine gate (cyan) and is believed to stop conformational changes that would allow the substrate to pass into cavity 2 (magenta) and be effluxed. Adapted from PDB: 7A6C (P-gp)^[96]^ and 6FEQ (ABCG2)^[112]^

Recent cryo-EM structures of human P-gp have provided additional information about the substrate as well as inhibitor binding sites. The structures of inhibitor bound P-gp are of particular interest in the development of future modulators of P-gp in relation to MDR, oral bioavailability, and the ability to access reservoirs beyond blood-tissue barriers. Using a human P-gp complexed with the Fab fragment of the inhibiting antibody MRK-16, cryo-EM structures of complexes with bound substrates and inhibitors (vincristine, paclitaxel, elacridar, tariquidar, and zosuquidar) were observed in an occluded state, which is characterized by the kinking of TMs 4 and 10 that also brings the nucleotide-binding domains closer together^[112,113]^. These structures revealed not only a large hydrophobic binding pocket within P-gp, consistent with evidence from many biochemical studies, but also a vestibule and access tunnel formed by transmembrane helices 7, 8, 9, and 12. Whereas substrates only occupy the central hydrophobic pocket, it was found that two inhibitor molecules can bind simultaneously to P-gp [Figure 2A]^[112,113]^. While one inhibitor molecule binds to the central binding pocket of P-gp, the second binds allosterically in the adjacent vestibule area extending away from the binding pocket and perhaps further into a tunnel that accesses the cytoplasm. The presence of inhibitor molecules in the vestibule and/or access tunnel is thought to either sterically inhibit conformational changes involving TM9 to an outward-facing conformation or trap the P-gp molecule in high energy occluded conformation. 

With this in mind, inhibitors and/or prodrugs could be designed to exploit this feature of P-gp by occupying the binding pocket, vestibule, and/or access tunnel simultaneously or stabilizing high energy occluded state through stabilization of TMHs 5, 7, 8, 9 and 12 at residues exposed to the access tunnel. Alternatively, inhibitors and or substrates could be joined by a linker moiety so that upon binding of P-gp, they would interact simultaneously with the binding pocket and extend into the access tunnel to stabilize a non-transporting conformation of P-gp. Several dimers of P-gp substrates and other molecules linked by variable-length tethers act as potent inhibitors and may be functioning to block P-gp function in such a manner^[84,86,142-154]^. The structures of P-gp with multiple inhibitors bound in the binding pocket and the vestibule provide interesting insights for strategies into inhibitor design. For instance, it may be possible to append additional aromatic moieties onto the binding pocket inhibitor that extend into the vestibule and access channel in such a way as to mimic portions of the inhibitor in that channel. In this way, increased contact with the transporters and increased binding affinity could be achieved.

Advances in cryo-EM have also allowed for precise structural determination of the homodimer of human ABCG2^[124-127]^. The ABCG2 structures revealed two cavities for substrate binding. A central hydrophobic slit-like cavity (cavity 1) faces the cytoplasmic side of the membrane and binds the substrate. Cavity 2 is smaller with less hydrophobic character with access to the extracellular space, thus promoting substrate release following transport. These two cavities are separated by a “leucine plug” that undergoes conformational changes during the transport cycle. Further conformational changes shift transmembrane helices 5b, 5c, and 6a, exposing cavity 2 to the extracellular space.

Recent structures of inhibitor bound ABCG2 could lead to future rationally designed inhibitors to exploit critical features of the inhibited conformation of ABCG2. Recent studies with the Ko143-derived ABCG2 inhibitor MZ29 demonstrated inhibition of ABCG2 occurs via binding of two molecules in cavity 1 and subsequent stabilization of the inward-facing conformation, thus not allowing the substrate to move across the membrane^[124]^. Additionally, structures with the tariquidar analog MB136 bound only one molecule of the inhibitor in cavity 1, and stabilized the transporter in the inward-facing conformation [Figure 2B]. Corroborative biochemical data also demonstrated that stoichiometric ratios of 1:1 for ABCG2:MB136 and 1:2 for MZ29 were needed for complete inhibition of ATPase activity. These inhibitors showed additional interactions and contacts with the transporter itself, including additional interactions in TM 1b, 2, and 5a that are not observed with molecules that are substrates for the transporter. It appears that the shape, size, and ability to fill the cavity to make these additional contacts are some of the critical features that discriminate inhibitor molecules from substrates, allowing them to form a tightly bound wedge to immobilize the transporter^[124,125]^. More recently, the anti-cancer drug imatinib was also found to lock the inward open structure of ABCG2^[127]^. In this study, one molecule of imatinib bound directly under the leucine plug in cavity 1 and was sandwiched between the interface of the ABCG2 monomers, similarly acting as a wedge to stabilize the inhibited, inward-facing conformation. These structural insights into how substrates and inhibitors interact with ABCG2 should be invaluable in designing future inhibitors, giving chemists an excellent platform for rational structure-based drug design of new inhibitors with higher binding affinities relative to substrates exploting these additional contacts in the TM domains.

## INHIBITOR DESIGN AND EVALUATION

After the discovery of P-gp, some substrates that could act as inhibitors were identified, including amiodarone, cyclosporine A, quinidine, and verapamil, and implemented in chemotherapy regimes in clinical trials^[3,17,19,23,24,27,29,57,58,155]^. The promising results observed in *in vitro* cell culture models were not replicated in patients, as this first generation of inhibitors, though pharmacologically active, was not potent and potentially toxic [Table 1]^[3,17,23,155,156]^. Second generation inhibitors that lacked pharmacological activity, such as valspodar and dexverapamil, were more potent than the first generation but resulted in no better outcomes due to off-target effects^[23,24,155-157]^. Third generation inhibitors including biricodar, elacridar, dofequidar, tariquidar, and zosuquidar were designed to have higher specificity for P-gp and lower toxicity in the hopes of better outcomes with co-administration with chemotherapeutics^[23,155,156]^. Tariquidar was shown to reverse drug resistance to doxorubicin caused by P-gp upregulation in a mouse model of breast cancer^[158]^. However, in a human clinical trial in non-small cell lung cancer patients, co-administration of tariquidar with chemotherapy led to increased toxicity and a halt to the study^[19,29]^.

**Table 1:** Representative inhibitors of P-gp and ABCG2

First generation	Cyclosporin A^*^, Verapamil^*^, Nifedipine^*^^,^^†^, Quinidine^*^, Quinine^*^, Tamoxifen^*^, Reserpine^*^^,^^†^, Prazosin^*^^,^^†^, Propafenone^*^^,^^†^, Erythromycin^*^, Itraconazole^*^^,^^†^, Ritonavir^*^^,^^†^, Benzquinamide^*[23]^
Second generation	Dexverapamil^*^, MM36^*^, Valspodar^*^^,^^†^, BIBW22BS^*^, Toremifene^*^, Quinine Homodimer (Q2)^*^, S9788^*,†[23]^
Third generation	Elacridar^*^^,^^†^, Tariquidar^*^^,^^†^, Zosuquidar^*^, Laniquidar^*^, Ontogen^*^, DP7^*[23]^
Fourth generation	Natural product derivatives: Flavonoids (Curcumin^*[163]^, Apigenin^*[164]^), Polyphenols (Quercetin^*[165]^, Resveratrol^*,†[166]^), Antimalarials (Chloroquine^*,†[167]^), Alkaloids (Galegine^*[168]^, Nitensidine A^*[169]^), Fungal (Ko143^†[170]^, Tryprostatin A^†[171]^), Chinese herbal medicines (Artemisinin^*[172]^, Tetrandrine^*[173]^), Marine (Kendarimide^*^ A, Sipholenol A^*^)^[174] ^ Repurposing FDA-approved drugs^[175]^: Imatinib^*^^,^^†^, Vardenafil^*^, Azithromycin^*^^,^^†^, Chlorpromazine^*^^,^^†^ Peptidomimetics^[23]^ Dual activity ligands^[23]^

^*^P glycoprotein inhibitor; ^†^ABCG2 inhibitor.

Additionally, and perhaps even more importantly, this trial was also seemingly flawed in that there is inconclusive evidence that non-small cell lung carcinomas express P-gp or that P-gp expression correlates with outcome clinically^[29,159]^. Further, once thought to be specific only for P-gp, tariquidar has also been shown to inhibit ABCG2, complicating interpretation of the results, perhaps partially accounting for the increased toxicity^[160,161]^. Thus, there remains room for improvement for even the best of the third generation inhibitors such as tariquidar. To that end, the structures of both ABCG2 and P-gp solved in the presence of tariquidar or a tariquidar analog^[112,124]^ offer a great opportunity to determine how differences in binding of the same molecule to the two different transporters may be exploited to generate even more specific inhibitors with higher affinity, and less cross-reactivity. Fourth generation inhibitor design is focused on natural product derivatives, peptidomimetics, dual activity ligands, and the potential of repurposing current FDA-approved drugs (see below for further discussion)^[23,162]^.

Due to the clinical importance of this area, many small-molecule inhibitors and modulators have continued to be developed for P-gp and ABCG2^[23,24,176-180]^. These designs focus on different aspects of blocking transport through either direct interaction with the drug binding site(s) or interaction with allosteric sites. To this end, different classes of inhibitors are being investigated including, but not limited to, dimeric substrates^[84,86,142-150]^, aromatic or heterocyclic dimers^[181,182]^, bifendates^[85]^, condensed ring derivatives^[183-185]^, 1,4-dihydropyridines and 1,4-dihydroquinolines^[186-188]^, dimeric flavonoids^[151-154]^, peptides^[189,190]^, steroids^[191-193]^, and tetrahydroisoquinolines^[194-196]^. For example, homodimers of the P-gp substrate quinine were potent inhibitors of P-gp and were found to reverse the drug resistance phenotype for taxol in a breast cancer cell line overexpressing P-gp^[149]^. In addition, homodimers of the P-gp substrate quetiapine demonstrated inhibition of P-gp in human brain capillary endothelial cells, in rat brain capillaries, and at the BBB in an *in situ* rat brain model^[144]^. Flavonoid dimers linked by polyethylene glycol units have also been shown to be inhibitors of P-gp^[151-153,197]^. Dual inhibitors of P-gp and ABCG2 were also developed using this strategy; homodimers of paliperidone with dual inhibition in a blood-brain barrier cellular model have been reported.

Interestingly, these homodimers, as well as dimers of abacavir, were designed to also revert to the monomeric drug within the reducing environment of the cell, thus acting as both ABC transporter inhibitors and prodrugs^[84,146]^. Recently, homodimers of triazole-bridged flavonoids were also shown to potently inhibit ABCG2 in a highly selective manner over P-gp^[154]^. These data suggest that exploiting the polyvalent nature of the drug binding sites of P-gp and ABCG2 may be an effective strategy for developing potent inhibitors of these transporters. Interestingly, as described above, structural studies demonstrated that several inhibitors were shown to bind in pairs to ABCG2 and P-gp^[112,113,124,125]^, suggesting that these dimeric inhibitors may have similar binding mechanisms. Thus, new inhibitor-bound structures of P-gp and ABCG2 should provide a more comprehensive understanding of inhibition, leading to better structure-based rational drug design for additional P-gp and ABCG2 inhibitors.

Another interesting prospect for inhibitor development is repurposing already FDA-approved drugs known as modulators P-gp and ABCG2 activity to improve the disposition of cancer chemotherapeutic drugs or, perhaps, agents used to treat other disorders^[175]^. These agents are also of particular interest as they can potentially provide both modulations of ABC transporters as well as their original FDA-approved function. As these FDA-approved drugs have already been rigorously tested clinically, have well-understood toxicities, and established mechanisms of action, co-administration with a chemotherapeutic drug may offer clinical benefits, barring unforeseen drug-drug interactions and changes in the pharmacokinetic and pharmacodynamic properties of either agent. In search of the Drugbank database of FDA-approved drugs, Lai *et al.*^[175] ^determined a set of 67 agents that have activity against P-gp but do not have anti-cancer activity. Interestingly, this list includes the anti-emetic/antipsychotic chlorpromazine^[198]^, the antibiotic macrolides erythromycin, azithromycin, and clarithromycin^[199]^, and the phosphodiesterases inhibitors sildenafil and vardenafil^[200]^.

Repurposing anti-cancer agents that are also P-gp and ABCG2 modulators have recently emerged as an area of more intense investigation, especially for tyrosine kinase inhibitors^[201]^. These agents, including elacridar, imatinib, sorafenib, dasatinib, gefitinib, nilotinib, erlotinib, and afatinib, are used in the treatment of a variety of cancers, including chronic myelogenous leukemia, gastrointestinal stromal tumors, renal cell carcinoma, and non-small cell lung cancer^[80,202-207]^. As these are also known modulators of P-gp and/or ABCG2, they could be used as chemosensitizers combined with other cytotoxic agents to increase the treatment efficacy. Care must be taken, however, as the inclusion of these agents as P-gp or ABCG2 inhibitors can also affect the pharmacodynamics and pharmacokinetics of any co-administered chemotherapeutic drug. For example, the addition of nilotinib in a mouse xenograft model of BCR-ABL chronic myelogenous leukemia treated with doxorubicin resulted in an increase in the cardiotoxicity of doxorubicin^[207]^, likely due to the inhibition of P-gp. Other anti-cancer agents being explored for repurposing as P-gp and ABCG2 inhibitors include PARP inihibitors^[208,209]^, CDK4/6 inhibitors^[210,211]^, and taxanes^[212,213]^.

## OTHER INHIBITORY APPROACHES

siRNAs downregulate protein expression by binding mRNA and forming RNA-induced silencing complexes recognized and digested by dicer RNases. Such siRNAs have been developed specifically for MDR1 or ABCG2 to reduce transporter expression and thus the MDR phenotype in tumor cells in culture^[214-216]^. In one study, siRNAs downregulated P-gp mRNA expression in drug-resistant breast carcinoma and osteosarcoma cell lines^[216]^. Further, transfection of the siRNA appeared to sensitize osteosarcoma cells to the anti-proliferative effects of doxorubicin, decreasing the IC_50_ 10-fold. In another study, transfection of drug-resistant NIH3T3-MDR fibroblasts with P-gp-targeted altritol-modified siRNAs resulted in a reduction of P-gp expression, a parallel reduction in P-gp mRNA levels, an increased accumulation of the P-gp substrate rhodamine 123, and increased sensitivity to doxorubicin^[214]^. Similarly, an ABCG2-targeted siRNA transfected into drug-resistant human liver HepG2 cells showed reduced levels of AGCG2 mRNA and increased sensitivity to doxorubicin, a known substrate of ABCG2^[215]^.

miRNAs are short noncoding RNAs, approximately 20-25 nucleotides, that modulate gene expression by binding the 3’ untranslated region (UTR) of target mRNA and have been explored as modulators of MDR in cancer cells. Expression of the miRNA miR-296 was shown to affect survival and MDR in esophageal squamous cell carcinoma^[217]^. In this study, cells treated with antagomirs demonstrated increased survival due to decreased tumorigenesis. Further, it was shown that expression of P-gp decreased, as measured by immunoblotting and RT-PCR, and cells were more sensitive to treatment with therapeutics known to be P-gp substrates^[217]^. In another study, over-expression of the miRNA miR-298 was shown to increase doxorubicin accumulation at the nucleus of resistant breast cancer cells through downregulation of P-gp expression via binding at the 3’ untranslated region of the MDR1 gene^[218,219]^. Downregulation of miR-298 in sensitive breast cancer cells showed increased expression of P-gp and conferred resistance to doxorubicin^[218]^. Transfection of miR-298 into human brain microvascular endothelial cells downregulated surface expression of P-gp and increased accumulation of antiepileptic drugs known to be substrates of P-gp^[219]^. miR-298 binding of the 3’ UTR of MDR1 was confirmed by cotransformation of P-gp non-expressing HEK293T cells with plasmids containing either the 3’ UTR of MDR1, miR-298, or controls and quantified through luciferase assay^[219]^. These studies demonstrate the potential utility of miRNAs in downregulating P-gp expression, either combat MDR, increase the bioavailability of P-gp substrates or allow the entry of therapeutic agents into sanctuary sites protected by P-gp, such as the brain.

Antisense oligonucleotides, which are short DNA or RNA sequences designed to bind target mRNA, provide another strategy to inhibit the expression of P-gp and ABCG2 at the translational level. Antisense oligonucleotides were shown to reduce the amount of MDR1/mRNA, and P-gp expression in human chronic myelogenous leukemia cells (K562) expressing P-gp^[220]^ and were also shown to increase doxorubicin accumulation in human glioblastoma and endothelial cell lines expressing P-gp^[221]^.

Gene manipulation using the CRISPR-Cas9 gene-editing technology is a newer approach that also affords an opportunity for treating drug-resistant cancers caused by ABC transporter expression or overexpression. For example, the CRISPR-Cas9 system could be used for downregulating or inactivating the genes that encode the ABC transporters implicated in multidrug resistance, such as MRP1, P-gp, and ABCG2, before treatment with chemotherapy^[222]^. A proof-of-concept experiment was performed successfully in a doxorubicin-resistant human epithelial ovarian cell line A2780/ADR, in which chemosensitivity to doxorubicin was restored following CRISPR-Cas9 knockdown of the human MDR1 gene that encodes P-gp^[223]^. CRISPR-Cas9 was also used in an MDCK canine kidney cell line to knock out endogenous P-gp expression. This new cell line could then be used to study exogenously expressed human or other P-gp’s, without the confounding effects of endogenous P-gp expression^[224]^. Using CRISPR-Cas9 to knock down ABC drug transporter expression may also be useful at the BBB where endogenous ABC transporters are of concern in limiting drug bioavailability to the brain. However, as permanent changes made by the CRISPR-Cas9 system to the expression of ABC transporters at the BBB may not be advantageous or even desirable, RNA editing techniques may offer more tunable systems for modulating the expression of these transporters in the future.

Monoclonal antibodies also have the potential to reverse MDR by binding to P-gp. MRK16, for example, was developed in the 1980s as a possible way to reverse drug resistance^[225-228]^. MRK16, which recognizes external epitopes of P-gp, was shown to work in concert with cyclosporin A treatment, operating synergistically to increase the accumulation of vincristine and doxorubicin in human leukemia K562 cells^[229]^. In another study, MRK-16 treatment was shown to partially restore sensitivity to vincristine-resistant tumor cells both *in vitro* and in a mouse xenograft model *in vivo*^[230]^. Furthermore, the monoclonal antibody UIC2 potently reverses P-gp-mediated drug resistance in cells in culture and recognizes extracellular epitopes of P-gp^[231,232]^. Interestingly, UIC2 was later determined also to be conformationally sensitive, binding to multiple conformations of the transporter^[231,233-235]^. Most recently, the structure of a chimeric human-mouse P-gp in complex with zosuquidar and UIC2 revealed that the antibody recognizes a conformational epitope of ABCB1 that involves residues from TM1 and TM2 and the extracellular loops EL1, EL3, and EL4^[114]^, which is in agreement with previous biochemical data^[232,234,235]^. The structure also gives insight into how the antibody acts as a transport inhibitor. By clamping the external loops together, UIC2 prevents P-gp from moving to the outward-open conformation required to release substrate to the extracellular space^[114]^. Unfortunately, although antibodies have great potential for selective and tight binding to ABC transporters, this field has not seen significant advancement since these early reports.

## CONCLUSIONS

An extensive effort has focused on multidrug resistance in cancer over the past 60 years, leading to the discovery of the ABC transporter superfamily. While the role of ABC transporters, including P-gp and ABCG2, has been studied extensively *in vitro*, there is still much to learn about the clinical importance of ABC transporter expression in cancer. While the early discovery of P-gp and ABCG2 inhibitors led to high expectations for clinical use, an FDA approved agent targeting P-gp or ABCG2 for combating multidrug resistance has been elusive, confounded by both other mechanisms of drug resistance and poorly designed clinical trials. Major advancements in structural biology in the last decade have led to valuable insights into the structures of human P-gp and ABCG2, and more recently, inhibitor-bound structures. These understandings will hopefully lead to more potent and specific inhibitors that, combined with more selective and rigorous clinical trials, lead to a deeper appreciation of the role of these transporters in the MDR phenotype in patients. Further, these inhibitors may have a positive impact on drug delivery efforts to sanctuary sites protected by endogenous expression of these transporters such as the brain and offer more effective oral bioavailability at the gut for many classes of chemotherapeutics. 

## DECLARATIONS

### Copyright

© The Author(s) 2021.
